# Role of Bile Acids and Nuclear Receptors in Acupuncture in Improving Crohn's Disease

**DOI:** 10.1155/2022/5814048

**Published:** 2022-05-13

**Authors:** Jia-Cheng Shen, Qin Qi, Dong Han, Shi-min Liu, Rong Huang, Yi Zhu, Han-dan Zheng, Kan Gu, Huan-gan Wu, Hui-rong Liu

**Affiliations:** ^1^Shanghai University of Traditional Chinese Medicine, Shanghai 201203, China; ^2^Shanghai Research Institute of Acupuncture and Meridian, Shanghai 200030, China; ^3^Yueyang Hospital of Integrated Traditional Chinese and Western Medicine, Shanghai University of Traditional Chinese Medicine, Shanghai 200437, China

## Abstract

Nuclear receptors (NRs) are ligand-dependent transcription factors that regulate the transcription of target genes. Bile acids (BAs) can be used as effector molecules to regulate physiological processes in the gut, and NRs are important receptors for bile acid signaling. Relevant studies have shown that NRs are closely related to the occurrence of Crohn's disease (CD). Although the mechanism of NRs in CD has not been clarified completely, growing evidence shows that NRs play an important role in regulating intestinal immunity, mucosal barrier, and intestinal flora. NRs can participate in the progress of CD by mediating inflammation, immunity, and autophagy. As the important parts of traditional Chinese medicine (TCM) therapy, acupuncture and moxibustion in the treatment of CD curative mechanism can get a lot of research support. At the same time, acupuncture and moxibustion can regulate the changes of related NRs. Therefore, to explore whether acupuncture can regulate BA circulation and NRs expression and then participate in the disease progression of CD, a new theoretical basis for acupuncture treatment of CD is provided.

## 1. Introduction

Crohn's disease (CD) is a clinically common inflammatory bowel disease (IBD) whose main clinical manifestations include gastrointestinal symptoms (recurrent abdominal pain, diarrhea, intestinal obstruction, perianal abscess, and anal fistula), fever, anemia, and malnutrition [1]. In recent years, epidemiology shows that the incidence of CD presents an increase in trend, and the highest incidence is mainly in developed countries and cities such as Europe and North America [2]. The acceleration of urbanization will lead to the occurrence of CD, the epidemiology of China shows that the incidence rate of CD is about 1.05 to 1.24/100000 people, and the incidence showed a gradient change from east to west [3]. At present, the exact cause of CD has not been fully revealed, which may be related to diet, environment, heredity, immunity, intestinal flora, and external bacterial and viral infection [1, 4]. Studies have shown that 12% of CD patients have family genetic history [5], and genomics studies have shown that 13.1% of patients are derived from genetic genes [6]. Therefore, genetics alone cannot explain the pathogenesis of CD, which highlights the importance of other nongenetic environmental factors [7]. A study shows that when low-risk countries and regions adopt Western lifestyle, the incidence rate of CD is rising sharply [8]. In addition, study has also shown that smoking increases the prevalence of CD by two times, indicating that smoking is an important inducement [9]. At the same time, the use of drugs such as aspirin and nonsteroidal anti-inflammatory drugs (NSAIDS), the decrease of dietary fiber intake, and the increase of saturated fat will increase the risk of CD [10, 11]. At present, drug is the main treatment method of modern medicine, including induction and maintenance therapy. The choice of drugs depends on the severity of the disease and the response to the previous treatment; the most widely used drugs for CD include corticosteroids, immunosuppressants such as thiopurine and methotrexate, and biological agents such as anti-TNF drugs (infliximab, adalimumab, and certolizumab pegol) and antiadhesion molecule (vedolizumab) [12].

Although drug treatment has achieved satisfactory results, there are still some disadvantages. For example, long-term use will cause serious side effects such as infection, tumor, and drug tolerance, and some patients have poor curative effect and are difficult to accept [13]. Therefore, a safe and effective complementary replacement therapy is urgently needed. According to statistics, the use of traditional Chinese medicine (TCM) therapy, including acupuncture, moxibustion, and herbal medicine, is increasing in IBD [14]. In Europe and America, about 70% of IBD patients receive complementary and alternative drug treatment [15, 16]. Acupuncture and moxibustion have a long history of clinical use in China and have a unique curative effect in the treatment of IBD [17]. In recent years, the clinical efficacy and mechanism of acupuncture and moxibustion have been widely explored. Clinical studies have shown that acupuncture and moxibustion can significantly improve the symptoms of IBD patients, inhibit intestinal inflammation, and repair intestinal mucosal barrier [18, 19]. Animal experiments show that acupuncture has a positive effect on TNBS-induced CD model rats, such as improving the protein or gene expression of inflammatory factors (TNF-*α* and IL-1*β*) [20].

Nuclear receptors (NRs) play an important role in the development of CD; NRs can regulate a wide range of functions in the gut, such as nutrient absorption and transport, gut-liver communication, intestinal flora regulation, and so on [21–23], the signal transduction of NRs is closely related to the immune function of the intestine, and the imbalance of NR signal can cause intestinal immune disorder [24]. As an important signal regulator in the gut-liver circulation, NRs can regulate the absorption of bile acids (BAs) in the gut. BAs can not only promote the absorption of lipids and vitamins, but also regulate a variety of functions in the gut as a signal molecule. Intestine is equipped with a complex BA sensing mechanism to coordinate different intestinal functions and control the communication between the gut and other organs. The pioneering work of some researchers has solved the role of BAs as signaling molecules, which can activate specific receptors to regulate biological processes and trigger cell signaling pathways. BAs can activate NRs and plasma membrane-associated receptors of different cell types and cause physiological effects of BAs [25]. In view of the fact that the complex physiological functions of the intestine and BAs as signaling molecules induce a variety of signal networks, the effect of BAs on intestinal function deserves special attention. Therefore, this review is designed to explore the mechanism of NRs in CD in the gut-liver circulation of BAs and the regulatory effect of acupuncture.

## 2. BAs Gut-Liver Cycle, NR Family, and IBD

BAs is mainly synthesized in the liver and then reabsorbed via the gut; the reabsorbed BAs are recycled back to the liver that established the gut-liver cycle of BAs [26]. BAs synthesis mainly includes two ways of classic pathways and alternative pathways, wherein the classic pathway is the main pathway, *n* is the passage, and cholesterol is catalyzed by Cholesterol7*α*-Hydroxylase (CYP7A1) in hepatocytes into primary BAs [27]. In the alternative pathway, cholesterol is catalyzed by cholesterol 27*α*-hydroxylase to synthesize chenodeoxycholic acid (CDCA). Primary BAs and CDCA combine with glycine or taurine to form conjugated primary BAs [28].

**Figure 1:**
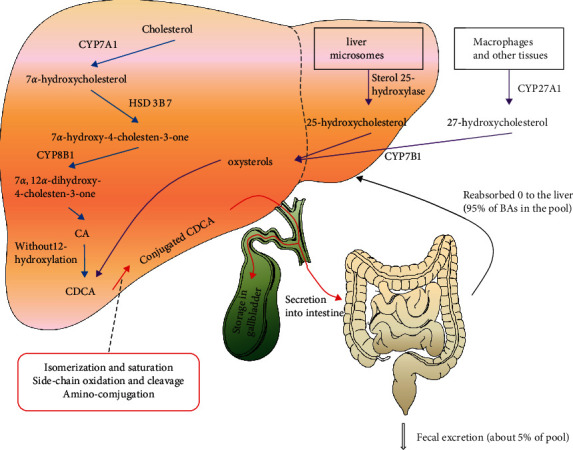
Schematic diagram of bile acid synthesis pathway and gut-liver circulation. The synthesis of BAs is mainly through classical and alternative pathways. The classical pathway is initiated by CYP7A1, and the alternative pathway is initiated by CYP27A1. CDCA is synthesized by cholesterol through a series of enzymatic reactions. CDCA forms conjugated CDCA by binding with glycine or taurine and are secreted into the intestine through bile duct. About 95% of BAs is transported into portal vein by intestinal epithelial cells and reabsorbed by hepatocytes and the remaining 5% will be excreted with feces. CYP7A1: cholesterol 7*α*-hydroxylase; HSD3B7: 3*β*-hydroxysteroid dehydrogenase; CYP8B1: sterol 12*α*-hydroxylase; CA: cholic acid; CDCA: chenodeoxycholic acid; CYP7B1: oxysterol 7*α*-hydroxylase; and CYP27A1: sterol 27-hydroxylase.

The conjugated primary BAs follow the secretion of bile into the intestine through the bile duct and participate in the absorption of lipids and fat-soluble vitamins. Under the action of intestinal microorganisms, the conjugated primary BAs undergo a series of dehydration and dehydroxylation to form secondary Bas. About 95% of the BAs enter the intestinal mucosal cells through the apical sodium-dependent bile acid transporter (ASBT) in the intestine and combine with ileum bile acid-binding protein (IBABP) to pass through the organic solute transporter *α*/*β* enters the portal vein [18], and under the mediation of Na+/taurocholate cotransporting polypeptide (NTCP) and organic anion transporting polypeptides (OATP), it is taken up again by hepatocytes and reentered into the intestine through the bile duct through the action of the bile salt export pump to form a complete BAs gut-liver circulation [29], while the remaining 5% will be excreted with feces (Figure 1) [30, 31].

The gut-liver circulation of BAs plays an important role in maintaining the normal level of BAs and the steady state of intestinal function that can not only increase the utilization of bile acids, promote the absorption of lipids and fat-soluble vitamins, but also inhibit the catalytic effect of CYP7A1 [32]. A large number of studies in the past few decades have shown that BAs not only participate in the absorption of lipids and fat-soluble vitamins in the intestine, but also act as effect molecules to regulate the function of the intestine [25]. As the main place of BAs circulation, the relationship between BAs and intestine has been widely studied. Intestine has a complete and complex response mechanism for BAs that BAs activate specific receptors in the intestine to further initiate intestinal-related signals pathways, including glucose and lipid metabolism and energy balance [33]. Among them, the important receptors that receive BAs activation signals in the intestine are the NR family [34].

**Figure 2:**
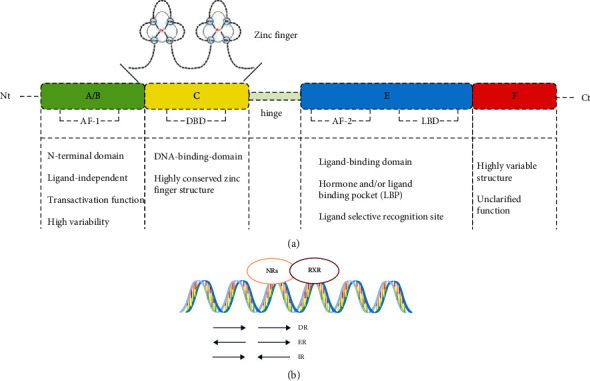
The general structure of nuclear receptors (a) and typical NR dimer (b). The main replication methods of NRs include direct repeat (DR), everted repeat (ER), and inverted repeat (IR). Nt: N-terminal; Ct: C-terminal; AF-1: activation domain 1; DBD: DNA-binding domain; AF-2: transcriptional activation domain; LBD: ligand-binding domain; and RXR: retinoid X receptor.

NRs are a type of ligand-dependent transcription factors that can be used to activate or inhibit the expression of target genes. So far, 48 NR family members have been found in the human body, which are important transcriptional regulators in the human body [35]. NRs can generally be divided into classic NRs and orphan receptors; about 50% of NRs belong to typical NRs [36]. Classical NRs mainly combine fat-soluble and membrane-permeable ligands and can be activated by endogenous BAs, hormones, or exogenous drugs or intestinal microbes [35, 36]. Classical NRs have similar structures, and their typical structures mainly include A/B, C, D, E, and F [33], the N-terminal A/B region is composed of active ligand-independent activation domain 1 (AF-1), and the A/B region is highly variable and generally contains at least one AF-1, C region belongs to DNA-binding domain (DBD), which has two zinc finger structures and characterized by being highly conservative [37]. The E region is a ligand-binding domain (LBD), which mainly plays the role of ligand recognition; its sequence is also highly conservative. It contains a ligand-dependent transcriptional activation domain (AF-2). The E region is the largest domain in NRs, which plays an important role in the selective recognition of ligands. There is a short and nonconservative hinge region between regions C and E, namely, region D. The C-terminal also contains a section of F-zone, whose structure height is variable, and the specific structure and function are not fully understood at present (Figure 2(a)) [38, 39]. The structure of orphan receptor is similar to that of classical NRs, but its physiological ligand is not clear at first [40].

Western diet characterized by high sugar, high fat, and low fiber intake is currently considered to be related to the incidence of CD [41]. Enteral nutrition is one of the confirmed induction therapies [42]. BAs are synthesized by the liver and stored in the gallbladder. After eating, they are released into the intestine under the stimulation of food, participating in food digestion and regulating the related functions of the intestine [43]. Studies have shown that CD patients will have poor absorption of bile acids, 95% of which will be recovered by reabsorption, and poor absorption of bile acids will lead to diarrhea and other symptoms [44]. With the abnormal changes of apical sodium dependent bile acid transporter (ASBT), breast cancer-related protein (BCRP), and fibroblast growth factor 19 (FGF-19), the abnormal expression of these factors will lead to increased synthesis, reduced transport and metabolism of bile acids, and excessive accumulation of bile acids in the intestine [45]; the increase of bile acid concentration is related to the structural change and permeability of intestinal mucosal barrier and can cause diarrhea [46]. Relevant studies have also shown that in patients with IBD, abnormal changes will occur in total BAs and other forms of BAs (e.g., combined BAs and glycocombined BAs) [21].

NRs are widely distributed in human body and play an important role in normal physiological functions, such as homeostasis, metabolism, growth, and development [47]. Current thinking suggests that NRs play a role mainly by interacting with other transcription factors and regulating the transcription of target genes [48]. With the development of research, it has been found that NRs, in addition to the above regulatory functions, play an important role in the progress of intestinal inflammation and intestinal mucosal barrier function in IBD [49–51]. At present, in vitro and in vivo models have proved that the expression of PXR and FXR in IBD is reduced, and the upregulation of PXR and FXR expression can improve intestinal inflammatory lesions [52]. Knockout of FXR or PXR promotes intestinal inflammation but does not induce spontaneous colitis [53]. Wilson et al.'s study showed that CD patients had abnormal composition of plasma bile acid spectrum, so PXR and FXR could not be activated normally [52]. Nijmeijer's group demonstrated that the target gene SHP expression of FXR decreased by 50% in CD patients, indicating the decreased activity of FXR [54]. Therefore, it is of great significance to consider the interaction between BAs and NRs in the treatment of CD.

The NRs involved in the progress of gastrointestinal inflammation and intestinal mucosal barrier function mainly include farnesoid X receptor (FXR) [55], pregnane X receptor (PXR) [56], vitamin D receptor (VDR) [57], constitutive androstane receptor (CAR) [58], peroxisome proliferator-activated receptor *γ* (PPAR*γ*) [59], retinoic acid-related orphan receptor *γ* (ROR*γ*) [60], and hepatocyte nuclear factor-4*α* (HNF-4*α*) [61]. BAs can activate the above NRs, participate in a variety of intestinal signaling pathways, and then regulate the intestinal function.

## 3. Regulating the Role of Members of NRs Family in CD Disease

Recent studies have shown that the NRs closely related to the progress of intestinal inflammation and intestinal mucosal barrier function of CD mainly include FXR, PXR, VDR, and CAR [62, 63]. FXR is also called bile acid receptor because it was confirmed to be the endogenous receptor of BAs at the beginning of its discovery [34]; it is expressed to varying degrees in tissues and organs such as liver, intestine, heart, blood vessel, kidney, and fat [64]. After FXR binds to the ligand, it can bind to the retinoid X receptor (RXR) to form an FXR/RXR dimer (Figure 2(b)), which can regulate the transcription of target genes [65]. The most important function of FXR is to adjust the balance of BAs [66, 67] and involved in the regulation of a wider range of physiological functions, such as metabolism, growth, and development of the human body [33]. With the development of genetic manipulation techniques, FXR^−/-^ mice have been widely used to study the mechanism of FXR in recent years [68, 69]. In the IBD model induced by dextran sulfate sodium (DSS) or 2,4,6-trinitrobenzene sulfonic acid solution (TNBS), FXR^−/-^ mice are more susceptible to TNBS and DSS [70, 71], and the mRNA expression of inflammatory factors in the colon showed higher level [53]. When FXR agonists such as INT-747 were used, the colitis induced by TNBS or DSS was significantly inhibited, the inflammatory infiltration was reduced [55], the intestinal permeability was decreased, and the loss of intestinal goblet cells and mucin was restored [72]. Nijmeijer [73] showed that FXR could inhibit the overgrowth of intestinal flora, which protect intestine and maintain functional stability Inagaki [54] showed that the expression of FXR/RXR dimer in CD patients was lower than that in healthy people, which indicated that FXR activity was inhibited in CD patients. FXR regulates intestinal immune function through the expression of immune cells, especially innate immune cells [74]; in addition to the adjustment of the innate immune system, FXR can also protect the intestinal epithelial cell barrier. The research of Gadaleta et al. [49] shows that the activation of FXR in the intestine lowered the expression of associated proinflammatory factors such as IL-6, MCP-1, and IL-1*β* and retain integrity of intestinal epithelial barrier function. In vitro, FXR agonist INT-747 significantly downregulated expression of TNF-*α*, IL-17, and IFN-*γ* in peripheral blood monocytes, CD14+ monocytes, dendritic cells, and lamina propria monocytes of IBD patients; on the other hand, deoxycholic acid and GW4064 can inhibit the healing of intestinal epithelial damage by inducing the nuclear accumulation of FXR [75]. And FXR activator ursodeoxycholic acid (UDCA) promotes damage repair [76]. The above studies can show that in addition to regulating the balance of BAs in the intestinal-hepatic circulation, FXR can inhibit intestinal inflammation and repair the intestinal mucosal barrier during IBD disease.

PXR is highly expressed in the intestinal and liver in the human [77], mainly regulating gene transcription and expression of participating in drug transportation and metabolism [78]. Pregnenolone-16*α*-carbonitrile (PCN) is a specific PXR agonist in rodents, while rifaximin and rifampicin are PXR agonists in human [40]. Randomized controlled trials (RCT) have shown that rifaximin can effectively improve the 12-week clinical remission rate of CD patients, and its mechanism may be related to rifaximin's activation of PXR to regulate the intestinal symptoms of CD [79, 80]. At the same time, in vivo experiments showed that in PXR knockout mice, T cells were significantly overproliferated and had a higher level of CD25 expression than wild-type mice. The activation of PXR can inhibit T cell proliferation and CD25, IFN-*γ* expression. PCN activation of PXR can protect DSS-induced colitis that is due to the activation of phase II enzymes and efflux transporters (e.g., GSTa1, MDR1a and MRP2), which can reduce the proinflammatory cytokines IL-6, TNF-*α*, MCP-1, and IL-1*α* expression. However, in PXR^−/-^ mice, the protective effect of PCN was abolished. Mechanism, PXR activation inhibits the activation of TNF-*α* on proinflammatory NF-*κ*B [81]. Mencarelli et al. [82] used primary fetal colonic epithelial cells to find that rifampicin inhibits the expression of IL-6, TNF-*α*, and IL-8 and promotes the expression of TGF-*β* by inhibiting lipopolysaccharide- (LPS-) induced NF-*κ*B DNA-binding activity. At the same time, PXR agonists can promote mucosal injury healing and intestinal barrier repair [50, 83, 84].

VDR is a regulatory receptor of 1,25-dihydroxyvitamin D (1,25[OH]2 vitamin D3), which mainly plays a role in regulating metabolism, immunity, and tumor development in the human body [85]. Vitamin D is an important sterol derivative; its deficiency is closely related to the onset of IBD [86]. Studies have shown that decreased expression of vitamin D in plasma will lead to an increase in the recurrence rate of IBD [87]. In IBD, vitamin D plays an important and complex protective role, which can participate in the regulation of the immune system through immune cells such as T cells [86], macrophages [88], and dendritic cells [89], and is considered to be a regulator of the immune system. Animal experiments show that vitamin D deficiency can aggravate the colitis symptoms of IL-10 gene knockout mice. Vitamin D supplementation can improve diarrhea caused by IBD and prevent weight loss [90]. Studies have shown that vitamin D enhances the integrity of the intestinal tract, which is characterized by high expression of tight junction protein and transepithelial resistance, while VDR gene knockout destroys the integrity of the intestinal tract [91]. Intestinal epithelial cells' (IEC) specific VDR knockout mice showed more severe colitis and higher expression of TNF-*α*, IL-1*β*, and MCP-1 than wild-type mice. Meanwhile, vitamin D3 inhibited the surface expression of MHC-II complex antigen and costimulatory molecules and downregulated the production of many proinflammatory cytokines [92]. The deficiency of vitamin D can induce the production of IL-22, which is closely related to the occurrence of colitis. In animal experiments, when vitamin D is deficient, mice will have more severe colitis [93]. It has been reported that in VDR knockout mice, the production of IL-22 in innate lymphocytes and antimicrobial peptides is significantly higher than that in wild-type mice, which may be an independent regulatory effect of VDR deficiency [94]. A 3-month RCT study showed that 1,25[OH] D levels were significantly increased in remission IBD patients, while intestinal permeability was maintained [95]. VDR also plays a protective role in colitis by regulating intestinal microbiota. Lack of VDR in intestinal epithelium can lead to autophagy defects and affect microbial aggregation [57]. Clinical studies showed that compared with the healthy control group, the microbial community of CD and UC patients changed significantly after taking vitamin D early [96, 97].

CAR is a kind of exogenous NRs regulated by exogenous, endogenous, and steroid hormones [98]. Its main metabolic function is to remove endogenous and exogenous substances [99]. Its expression in intestine and liver is closely related to intestinal flora [100, 101]. CAR was expressed in healthy intestinal epithelium but decreased in UC and CD patients or DSS mice [58, 102]. In the preclinical DSS mouse model, especially in CAR-deficient mice, wound healing of intestinal epithelial cells was reduced, while the activation of CAR with selective CAR agonist 3,3′,5,5′-tetrachloro-1,4-bis(pyridyloxy)benzene (TCPOBOP) enhanced mucosal healing [58]. In DSS-induced colitis model, CAR agonist decreased the mRNA expression of several proinflammatory cytokines in a CAR-dependent manner. In in vitro cell analysis, CAR inhibited apoptosis by inducing Gadd45b [103].

## 4. Mechanism of Acupuncture Treatment of CD and Regulation of BAs and NRs

Because the etiology and pathogenesis of IBD are not completely clear, and there is no specific therapy at present, the main clinical treatment methods mainly come from Western medicine, including the traditional sulfasalazine (SASP; 5-aminosalicylic acid (5-ASA)), steroids, immunosuppressants, or biological agents. However, the long-term use of steroids or immunosuppressants will cause serious adverse reactions, and the biological agents are not only expensive so that they lead to a heavy economic burden, but also show unsatisfactory long-term efficacy [104]. As an important part of TCM, acupuncture and moxibustion play an important role in the treatment of many diseases based on the theory of meridians [105]; in clinic, acupuncture is often used to treat CD [106]. According to the theory of TCM, the incidence of CD is closely related to the liver (*Gan*), spleen (*Pi*), and kidney (*Shen*) of viscera. When the external or internal causes affect the viscera, or the spleen and kidney have defects, it will cause the symptoms of CD in the intestine, including damp heat accumulation (*shi re nei yun*), qi fixation and blood stasis (*qi zhi xue yu*), liver stagnation by spleen (*gan yu cheng pi*), spleen deficiency and dampness fixation (*pi xu shi kun*), and spleen and kidney yang deficiency (*pi shen yang xu*). Acupuncture and moxibustion have the functions of dredging meridians, anti-inflammatory and analgesic, warming meridians, warming and dispersing cold evil, eliminating swelling and knot, promoting blood circulation, and removing blood stasis. Clinically, it is commonly used to treat CD acupoints, including Taixi (KI3), Tianshu (ST25), Zhongwan (CV12), Guanyuan (CV4), and Zusanli (ST36) [107]. At present, the recognized mechanism of acupuncture and moxibustion in the treatment of IBD is that the vagus nerve regulates the immune response, mainly including 3 pathways: (1) cholinergic anti-inflammatory pathway (CAP), the stimulation signal of acupuncture and moxibustion reaches the intestinal nerve through the vagus nerve and the intestinal neurons release acetylcholine at the synaptic connection with immune cells *α*-7-nicotinic acetylcholine receptor binding has been the release of proinflammatory factor (TNF-*α*); (2) splenic sympathetic anti-inflammatory pathway, the splenic nerve releases norepinephrine after receiving the signal of vagus nerve, which is related to the function of splenic lymphocytes *β*2 adrenergic receptor binding, and then through *α*-7-nicotinic Ach receptor inhibits the release of TNF-*α* from splenic macrophages; (3) the hypothalamus pituitary adrenal (HPA) axis receives vagal stimulation, which causes the adrenal gland to release cortisol [108].

In clinical trials, the expression of tight junction protein in intestinal epithelium was upregulated after receiving mesalazine or acupuncture treatment in CD patients [18]. In another RCT study, the efficacy of acupuncture and moxibustion was compared with that of sham acupuncture, and this experiment showed that the CD activity index score (CDAI) of the acupuncture group was improved; in addition, compared with the sham acupuncture group, the expression level of IL-17 in the acupuncture group was decreased, while the T regulatory cells were increased [19]. In a study using similar sham acupuncture treatment to observe mild-to-moderate CD patients, the levels of CDAI, C-reactive protein, and hemoglobin were significantly improved after acupuncture treatment, but no significant difference was found in endoscopy between the two groups [109]. A study that observed acupuncture alone also showed a significant decrease in CDAI scores compared with the sham acupuncture group [110]. A meta-analysis about the efficacy of acupuncture and moxibustion for IBD, including 43 RCT study, has shown that acupuncture and moxibustion have a significant effect on IBD. 10 studies compared acupuncture and moxibustion with SASP, showing that acupuncture and moxibustion are better than oral SASP in the treatment of IBD [104]. This shows that acupuncture and moxibustion have a significant effect on CD, which is related to the downregulation of intestinal inflammatory indexes.

At the same time, BAs and NRs are closely related to the changes of intestinal immune function. Therefore, it is worth studying whether acupuncture and moxibustion can produce therapeutic effect on CD by regulating the expression of BAs and NRs. At present, the research on the regulation mechanism of acupuncture and moxibustion on BAs and NRs is very limited. Current research shows that acupuncture and moxibustion play a positive role in the regulation of bile acid metabolism. In studying the effect of herb-partitioned moxibustion on metabolites in IBS rats, Lin et al. [111] found that there is an obvious imbalance of bile acid metabolism pathway in IBS rats, mainly manifested in the reduction of bile acid transformation and reabsorption, which can be improved after herb-partitioned moxibustion treatment. Lee et al.'s [112] results also show that manual acupuncture can reduce the deposition of bile acids, which may be related to reducing the activation of spinal microglia. In the study of the regulation of herbs-partition moxibustion on the nuclear receptor LXR*α* in the reversal of cholesterol transport in atherosclerosis (AS), Wang et al. found that herbs-partition moxibustion can reduce total cholesterol and low-density lipoprotein in serum and liver of AS animal model and promote the synthesis of high-density lipoprotein. At the same time, herbs-partition moxibustion can activate the expression of LXR*α* protein and mRNA and promote the reverse transport of cholesterol. Cui et al. [113] also obtained similar results in the study of moxibustion in the treatment of ApoE^−/-^ mouse AS model. Moxibustion can regulate lipid metabolism and upregulate the expression of LXR*α* and ABCA1 to prevent lipid accumulation. The results published by Liu et al. [114] and Li et al. [115] showed that electroacupuncture preconditioning has a protective effect on rats with myocardial ischemia-reperfusion injury, and its mechanism is related to the regulation of FXR/SHP pathway. It is confirmed from the above limited research has confirmed that acupuncture and moxibustion can regulate the liver-intestinal circulation of cholesterol and participate in related pathways mediated by NRs.

**Figure 3:**
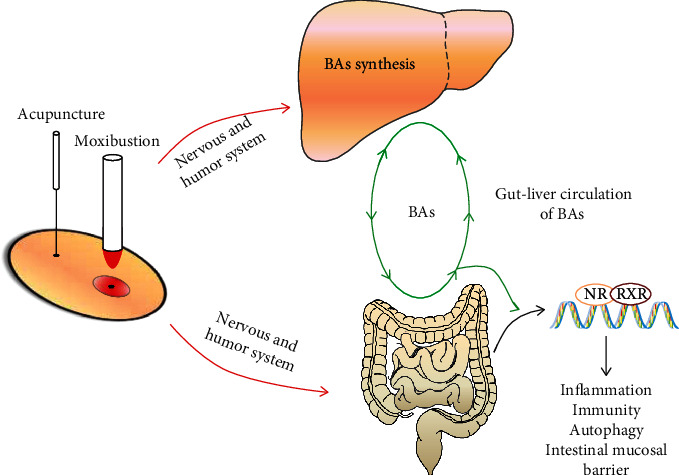
Effects of acupuncture and moxibustion on gut-liver circulation of BAs and the mechanism of regulating NRs.

Combined with the study of the therapeutic mechanism of acupuncture and moxibustion, and the related role of BAs-NRs in gut-liver circulation, we speculate that in the treatment of CD, acupuncture and moxibustion cause the increase of BAs synthesis in the liver by stimulating local acupoints and through the signal input of nervous and humor system. After BAs are released into the intestine, the NRs were activated and then play a role in intestinal immune regulation and intestinal mucosal barrier repair (Figure 3). The gut-liver circulation of BAs can maintain the balance of BAs and play an important role in promoting the BAs-NRs signal pathway. However, at present, in the treatment of CD, the mechanism on acupuncture and moxibustion of BAs-NRs is very limited, and more basic and clinical research support is needed.

## 5. Conclusion

CD is currently a refractory gastrointestinal disease in clinical practice, which belongs to one of IBD. Existing research evidence shows that BAs and NRs families play an important role in the progression of CD, which can inhibit the inflammatory progress of CD colon and promote the repair of intestinal mucosal barrier. The NRs involved in the progression of CD disease mainly include FXR, PXR, VDR, CAR, PPAR*γ*, and ROR*γ*. A large number of clinical and animal experiments have shown that NRs can participate in the pathogenesis of CD by regulating related signal pathways such as inflammation, immunity, autophagy, and intestinal mucosal barrier. Acupuncture is an important part of TCM. Both clinical practice and in vivo experiments show that acupuncture has obvious curative effects in the treatment of CD; at the same time, acupuncture can adjust the changes of related NRs. Therefore, exploring whether acupuncture and moxibustion can participate in the disease progression of CD by regulating BA circulation and the expression of NRs can provide a new theoretical basis for acupuncture and moxibustion in the treatment of CD.

## Data Availability

The data used to support the findings of this study are available from the corresponding author upon request.
